# Equation of State for Europium at High Pressures and Entropies in Shock Waves

**DOI:** 10.3390/ma19153351

**Published:** 2026-08-06

**Authors:** Konstantin V. Khishchenko

**Affiliations:** Joint Institute for High Temperatures of the Russian Academy of Sciences, Izhorskaya 13 Bldg 2, Moscow 125412, Russia; konst@ihed.ras.ru

**Keywords:** equation of state, europium, shock waves, high pressures, high entropies, high energy densities

## Abstract

This work is devoted to the description of the thermodynamics of the condensed states of the rare-earth metal europium under intense mechanical and thermal loading in shock waves. A new model of the equation of state for metallic materials is proposed based on the characteristic function of entropy with volume and internal energy (per unit mass) as variables. Within the framework of this model, calculations of the thermodynamic characteristics of europium at high pressures and specific entropies were carried out, the results of which are presented in comparison with the available data from shock-wave experiments. The developed equation of state for this material can be effectively applied to the modeling and simulation of various dynamic processes at high energy densities.

## 1. Introduction

To model and simulate the dynamic response of materials to intense impacts [1,2,3], it is necessary to have a closing relation for the system of equations of motion of matter. The equation of state, which expresses the relationship between the thermodynamic functions of a substance and thermodynamic variables, is traditionally used as such a closing relation [4,5,6,7,8]. It is clear that the adequacy of the equation of state to the available experimental data mainly determines the correspondence of the results of modeling and simulations to the ideas that exist about the laws of the processes under consideration [9,10,11,12,13].

Europium is characterized by a high thermal neutron capture cross-sections [14,15,16] and is used in nuclear power engineering as a neutron absorber. The equation of state for europium is in demand for the modeling and simulation of physical processes in nuclear equipment components operating under conditions of intense mechanical and thermal loads [17,18,19,20,21].

In a high-pressure region, europium has been studied both under dynamic conditions of shock-wave loading [22,23,24,25] and under static compression conditions at constant room (or elevated) temperature [26,27,28,29,30,31].

Known approaches [23,24] to constructing equations of state for this metal in shock waves are based on the function [23] of pressure (*P*) upon the specific volume (*V*) and specific internal energy (*E*),

(1) P=P(V,E),

or the functions [24] of internal energy upon the specific volume and pressure, E=E(V,P), and pressure upon the specific volume and absolute temperature (*T*), P=P(V,T).

In this study, the equation of state for a metallic material is proposed to be specified in the form of a function of specific entropy (*S*) upon specific volume and specific internal energy:

(2) S=S(V,E).

Then, according to the first and second laws of thermodynamics [32], written for reversible processes in the form of a differential,

(3) dE=−PdV+TdS,

which is rewritten in the form

(4) $$dS=T^{-1}PdV+T^{-1}dE,$$

an equation of state in form (1) can be obtained by differentiating expression (2):

(5) $$T^{-1}P={\left(\partial S/\partial V\right)}_{E},$$

and

(6) $$T^{-1}={\left(\partial S/\partial E\right)}_{V},$$

and then

(7) $$P={\left(\partial S/\partial V\right)}_{E}{\left[{\left(\partial S/\partial E\right)}_{V}\right]}^{-1}.$$

And while the caloric equation of state in form (1) can be used separately in dynamic modeling, obviously, only of adiabatic processes (for example, in shock-wave flows arising under the action of a high-velocity body [33,34,35,36] or a short high-intensity laser pulse [37] on a solid target), characteristic function (2) allows one to additionally obtain, by means of differentiating (6), a thermal equation of state in the form

(8) T=T(V,E),

which is in demand in the modeling of heat-transfer processes (for example, near the interface of colliding bodies [38] or during the interaction of radiation with matter [39,40,41]).

Recently [42], based on thermodynamic relation (3), an equation-of-state model for a metallic material (aluminum) was proposed in the form of a characteristic function of the specific volume upon the entropy and internal energy, V=V(S,E). It is also appropriate to mention an example of constructing equations of state for metals (copper and lead) in the canonical form of the thermodynamic potential of internal energy as a function of volume and entropy [43], E=E(V,S).

In this work, based on characteristic function (2) for europium, calculations of the thermodynamic parameters of condensed states under shock-wave loading were carried out, and the results of these calculations are presented in comparison with the available experimental data for this material at high pressures and specific entropies.

## 2. Equation-of-State Model

In the proposed model of the equation of state in form (2), the characteristic function of the specific entropy of the metal is given by the following expression:

(9) $$S\left(V,E\right)=\frac{3}{2}R\left(\ln\frac{{\left[\zeta_{v}\right]}^{2}}{1+\zeta_{v}}+\frac{1}{1+\zeta_{v}}+2\beta_{a}\zeta_{v}\right)+S_{1},$$

where *R* is the specific gas constant, $R=k_{B}{\left(A_{u}m_{u}\right)}^{-1}$; $k_{B}$ is the Boltzmann constant; $A_{u}$ is the relative atomic mass; $m_{u}$ is the atomic mass unit; $\beta_{a}$ is a dimensionless constant; $S_{1}$ is the constant of the dimension of entropy; $\zeta_{v}$ is a dimensionless function of volume and internal energy that is established using two other dimensionless parameters, $\xi_{v}$ and $\xi_{t}$. The relationship of $\zeta_{v}$ with $\xi_{v}$ and $\xi_{t}$ is given by means of two equations:

(10) $$\beta_{a}{\left[\zeta_{v}\right]}^{3}+\left(1+\beta_{a}\right){\left[\zeta_{v}\right]}^{2}+2\left(1-\xi_{v}\right)\zeta_{v}-2\xi_{v}=0$$

and

(11) $$\xi_{v}+\ln\left(\theta_{c}\xi_{v}\right)=\xi_{t}+\ln\left(\theta_{i}\xi_{t}\right),$$

where

(12) $$\xi_{t}\left(V,E\right)=\frac{E-E_{c}\left(V\right)}{\theta_{i}E_{a}},$$

$E_{c}$ is the specific internal energy on the isotherm T=0 (cold curve); $E_{a}$ is the constant of the dimension of specific energy; $\theta_{c}$ and $\theta_{i}$ are dimensionless functions of volume,

(13) $$\theta_{c}\left(V\right)=\sigma^{2/3}\exp\left[\left(\gamma_{0c}-2/3\right)\frac{\delta_{n}+{\left[\ln\sigma_{m}\right]}^{2}}{\sqrt{\delta_{n}}}\left(\arctan\frac{\ln\sigma -\ln\sigma_{m}}{\sqrt{\delta_{n}}}+\arctan\frac{\ln\sigma_{m}}{\sqrt{\delta_{n}}}\right)\right],$$

$\theta_{i}\left(V\right)=\sigma^{\gamma_{i}}$. Here, $\sigma =V_{0}/V$; $V_{0}$ is the specific volume under normal conditions ($E=E_{0}$ and $P=P_{0}$); $\delta_{n}$, $\sigma_{m}$, $\gamma_{0c}$, and $\gamma_{i}$ are dimensionless constants.

Cold energy is given in the form of the function

(14) $$E_{c}\left(V\right)=\frac{B_{0c}V_{0c}}{m-n}\left(\frac{\varsigma^{m}-1}{m}-\frac{\varsigma^{n}-1}{n}\right),$$

in which $\varsigma =V_{0c}/V$; $V_{0c}$ is the specific volume at T=0 and P=0; $B_{0c}$ is the bulk modulus at T=0 and ς=1; *m* and *n* are dimensionless constants.

The constant $S_{1}$ in (9) is selected from the condition $S\left(V_{0},E_{0}\right)=S_{0}$, which is expressed as follows:

(15) $$S_{1}=S_{0}-\frac{3}{2}R\left(\ln\frac{{\left[\zeta_{0}\right]}^{2}}{1+\zeta_{0}}+\frac{1}{1+\zeta_{0}}+2\beta_{a}\zeta_{0}\right),$$

where

(16) $$\zeta_{0}=\frac{3RT_{0}}{E_{a}},$$

$T_{0}$ is the normal temperature.

Relations (9)–(16) completely define the equation of state in form (2) for the metallic material under consideration. Thermodynamic functions (other than entropy) depending on the specific volume and specific internal energy can be found using differential relation (4). In particular, the expression for temperature (8) is obtained by differentiating relations (9)–(12) according to (6) and is transformed into the following form:

(17) $$T\left(V,E\right)=\frac{E_{a}}{3R}\theta_{c}\zeta_{v}\frac{1+\xi_{v}}{1+\xi_{t}}\exp\left(\xi_{v}-\xi_{t}\right).$$

The expression for pressure is obtained using relation (7) and takes the form of the caloric equation of state (1):

(18) $$P\left(V,E\right)=P_{c}\left(V\right)+\frac{\Gamma (V,E)}{V}\left[E-E_{c}\left(V\right)\right],$$

where $P_{c}$ is the pressure at T=0; the coefficient Γ is a dimensionless function of *V* and *E*,

(19) $$\Gamma \left(V,E\right)=\gamma_{i}+\frac{\gamma_{c}\left(V\right)-\gamma_{i}}{1+\xi_{t}},$$

in which $\gamma_{c}$ is the Grüneisen coefficient $\gamma =V{\left(\partial P/\partial E\right)}_{V}$ at T=0, $\gamma_{c}=d\ln\theta_{c}/d\ln\sigma$,

(20) $$\gamma_{c}\left(V\right)=2/3+\left(\gamma_{0c}-2/3\right)\frac{\delta_{n}+{\left[\ln\sigma_{m}\right]}^{2}}{\delta_{n}+{\left[\ln\sigma -\ln\sigma_{m}\right]}^{2}}.$$

Using (18)–(20), one can easily obtain the relation $\gamma_{0c}=\gamma_{i}+\left(\gamma_{0}-\gamma_{i}\right){\left[1+\xi_{0}\right]}^{2}$, where $\gamma_{0}$ is the Grüneisen coefficient under normal conditions;

(21) $$\xi_{0}=\frac{E_{0}-E_{c}\left(V_{0}\right)}{E_{a}}.$$

Cold pressure in (18) is obtained by differentiating relation (14), $P_{c}=-dE_{c}/dV$:

(22) $$P_{c}\left(V\right)=\frac{B_{0c}}{m-n}\left(\varsigma^{m}-\varsigma^{n}\right)\varsigma .$$

It should be noted that expressions (14), (18)–(20) and (22) for pressure as a function of volume and internal energy are close to expressions of the equation-of-state model [44,45,46].

As a limitation of the applicability range of model expressions (9)–(12), one can apply the condition of the smallness of the absolute temperature (17) compared to the Fermi temperature ($T_{F}$) [47]:

(23) $$T_{F}\left(V\right)=\frac{\hbar^{2}}{2m_{e}k_{B}}{\left(\frac{3\pi^{2}Z\sigma }{A_{u}m_{u}V_{0}}\right)}^{2/3},$$

where *ℏ* is the reduced Planck constant; $m_{e}$ is the electron mass; *Z* is the atomic number.

## 3. Thermodynamic Characteristics of Europium at High Pressures

**Figure 1:**
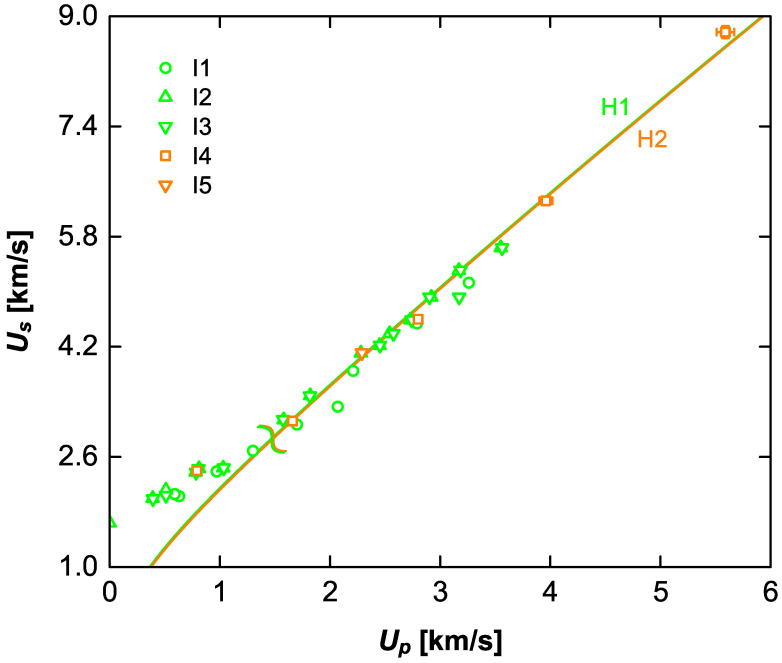
Shock velocity as a function of particle velocity on shock adiabats of samples with different initial densities ($\rho_{00}$) for europium: solid lines—the results of present calculations for $\rho_{00}=5.29$ (H1) and 5.19 g/cm^3^ (H2); markers—data from shock-wave experiments (I1—adapted from Ref. [23]; I2—adapted from Ref. [24]; I3, I5—adapted from Ref. [25]; I4—adapted from Ref. [22]); lines and markers of the same color correspond to the same initial density of samples; wavy lines show the approximate position of the lower boundary of the considered state regions on the shock adiabats.

**Figure 2:**
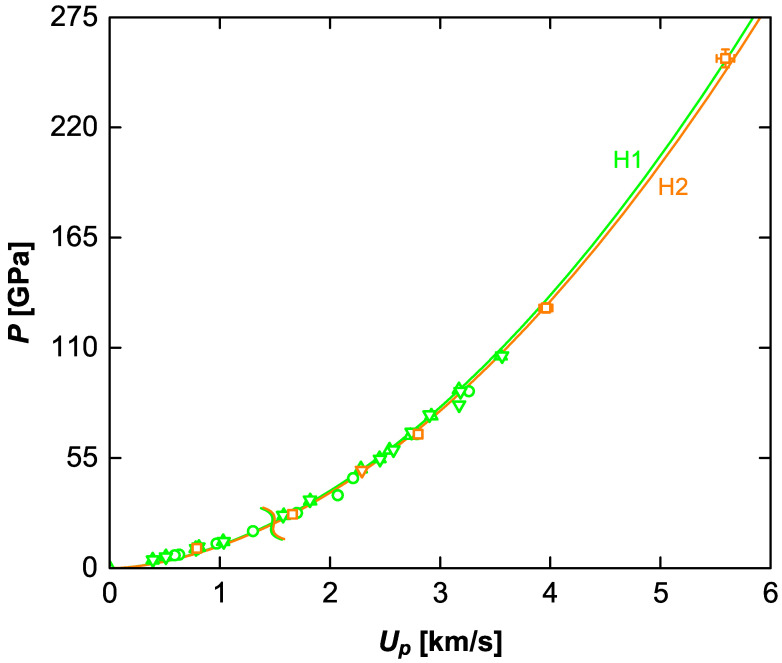
Pressure as a function of particle velocity on shock adiabats of samples with different initial densities for europium: the designations are the same as in Figure 1.

**Figure 3:**
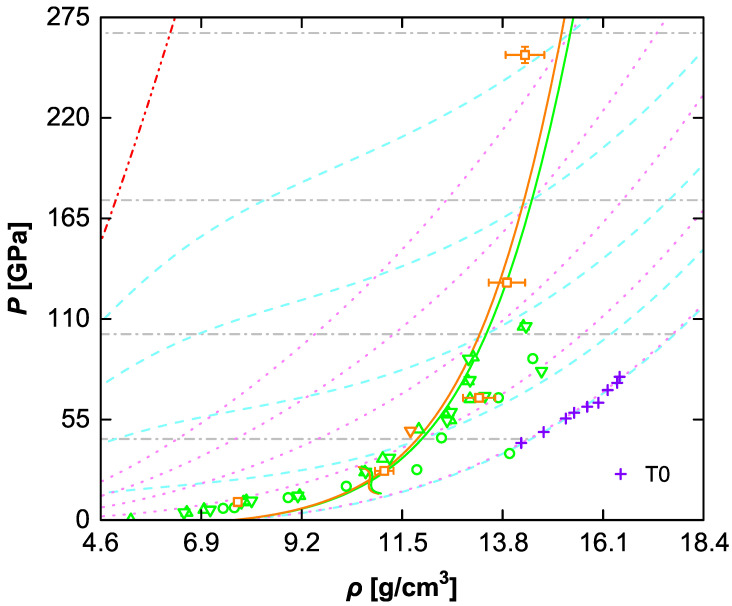
Diagram of states for europium in the considered range of values of density and pressure: lines—the results of present calculations for shock adiabats (solid lines) of samples with initial densities $\rho_{00}=5.29$ and 5.19 g/cm^3^ (for adiabats from right to left), isotherms (dashed lines) at T=0.293, 9, 18, 27, and 36 kK (for isotherms from bottom to top), isentropes (dotted lines) at S=0, 0.616, 0.832, 1.008, and 1.171 J/[g K] (for isentropes from bottom to top), and isobars (dash-dot lines) at P=44.3, 101.7, 175, and 266.4 GPa (for isobars from bottom to top), as well as for the curve at $T=T_{F}/10$ (dash-dot-dot line); crosses—data from isothermal-compression experiments at room temperature (T0—adapted from Ref. [31]); the rest of the designations are the same as in Figure 1.

**Figure 4:**
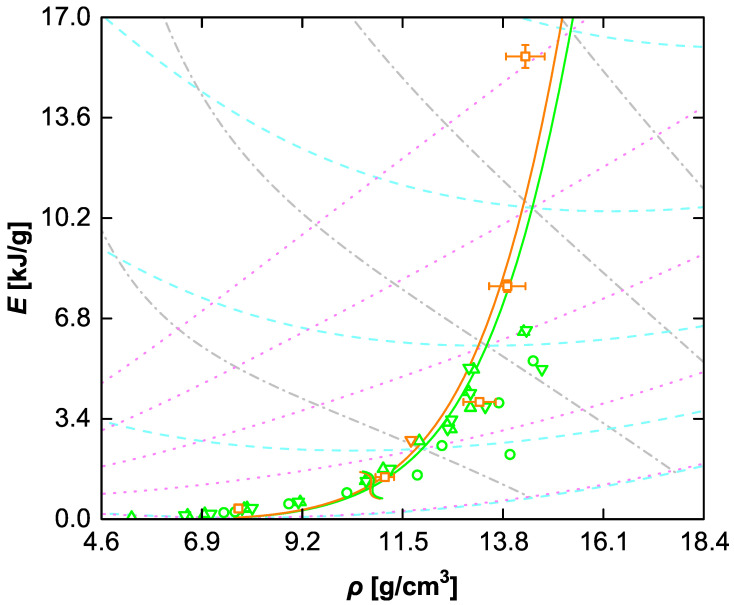
Diagram of states for europium in the considered range of values of density and specific internal energy: the designations are the same as in Figure 3.

**Figure 5:**
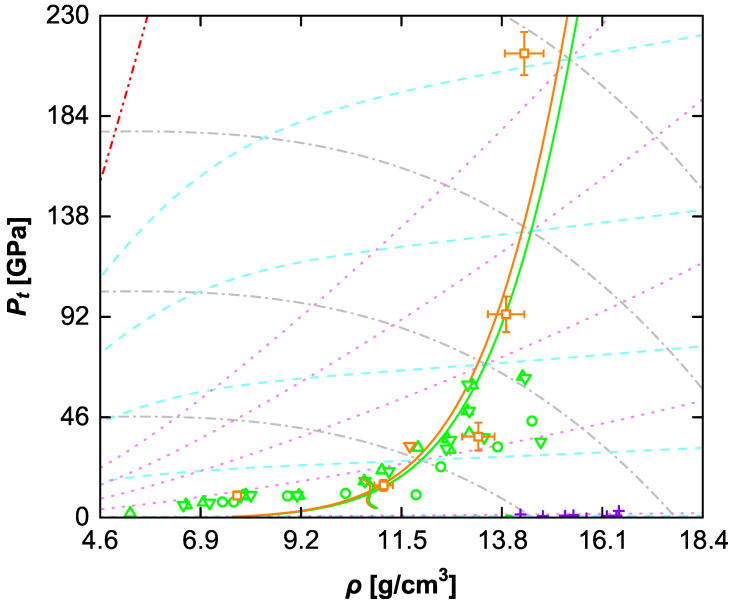
Diagram of states for europium in the considered range of values of density and thermal part of pressure ($P_{t}=P-P_{c}$): the designations are the same as in Figure 3.

**Figure 6:**
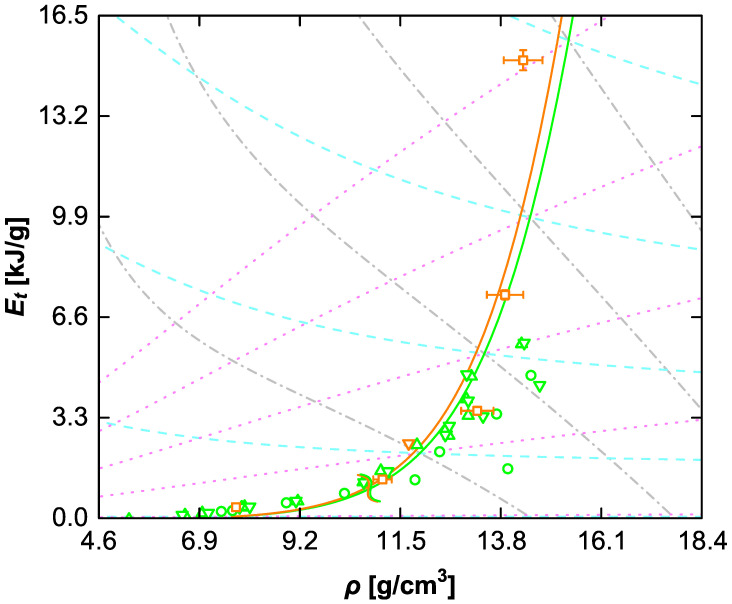
Diagram of states for europium in the considered range of values of density and thermal part of specific internal energy ($E_{t}=E-E_{c}$): the designations are the same as in Figure 3.

**Figure 7:**
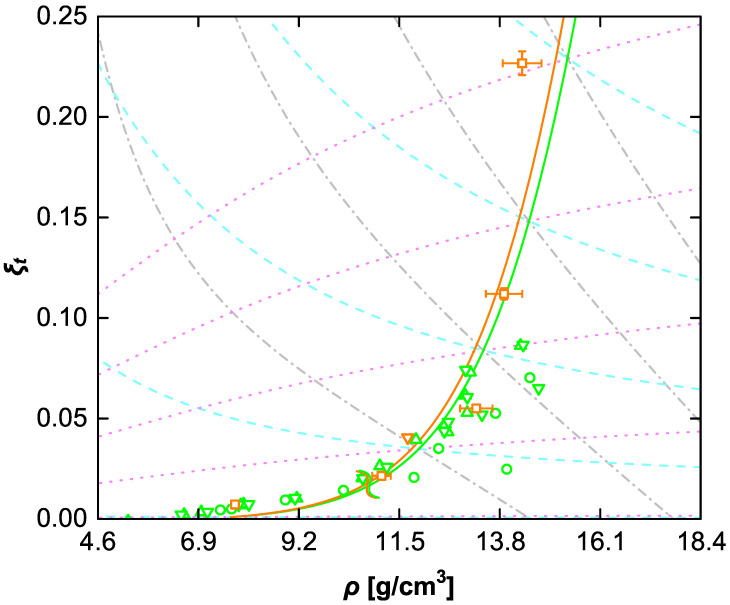
Diagram of states for europium in the considered range of ρ and $\xi_{t}$ values: the designations are the same as in Figure 3.

To illustrate the quality of the proposed equation-of-state model in describing the characteristics of the material under consideration at high pressures, the results of calculations of the shock adiabats for samples of initial densities $\rho_{00}=5.29$ and 5.19 g/cm^3^ are displayed in Figure 1, Figure 2, Figure 3, Figure 4, Figure 5, Figure 6 and Figure 7 in comparison with the available data from shock-wave experiments [22,23,24,25].

Calculations of shock adiabats were performed based upon the solution of the Hugoniot equation [1],

(24) $$E=E_{0}+2^{-1}\left(P+P_{0}\right)(V_{00}-V),$$

together with the caloric equation of state in form (1) relating pressure, specific volume, and specific internal energy within the framework of expressions (9)–(22). Here, $V_{00}=1/\rho_{00}$, $P_{0}=0.1$ MPa, and $P\left(V_{0},E_{0}\right)=P_{0}$. The velocity of the shock-wave front (shock velocity, $U_{s}$) and the velocity of the substance behind this front (particle velocity, $U_{p}$) were determined using the following relationships [1]:

(25) $$U_{s}=V_{00}\sqrt{\frac{P-P_{0}}{V_{00}-V}}$$

and

(26) $$U_{p}=\sqrt{\left(P-P_{0}\right)\left(V_{00}-V\right)}.$$

The diagrams presented in Figure 1 and Figure 2 reveal that there is good agreement between the calculated curves and the experimental points in the entire studied range of particle velocities above 1.5 km/s (and pressures of shock-wave loading above 20 GPa). At lower particle velocities (and lower pressures), the available data for europium from shock-wave experiments [22,23,24,25] indicate some phase transformation, the consideration of which is beyond the scope of this work.

The ranges of densities (ρ=1/V), pressures, and internal energies studied in shock-wave experiments [22,23,24,25] for europium are demonstrated in the diagrams of states in Figure 3 and Figure 4. The shock adiabats for samples with $\rho_{00}=5.29$ and 5.19 g/cm^3^ in these diagrams are in good agreement with experimental data [22,23,24,25] in the region corresponding to pressures above 20 GPa.

In addition to the shock adiabats, the calculated isotherms (T=0.293, 9, 18, 27, and 36 kK), isentropes (S=0, 0.616, 0.832, 1.008, and 1.171 J/[g K]), and isobars (P=44.3, 101.7, 175, and 266.4 GPa) are also shown in Figure 3 and Figure 4. The presented isolines characterize the values of temperature and specific entropy in the considered region of pressures and specific internal energies.

It is worth mentioning here that, under normal pressure conditions, europium has a body-centered cubic structure [48] and melts at T=1.095 kK [18,49].

An analysis of the results of calculating the pressure in the material at a temperature ten times lower than the Fermi temperature (23), which are presented in Figure 3, allows one to conclude that all states under consideration belong to the range of applicability of model expressions (9)–(12).

In Figure 3, high-pressure data for states of europium with a monoclinic structure from experiments [31] carried out at constant room temperature are shown as well. One can see that the calculated isotherm at T=0.293 kK is in good agreement with these data.

It should be noted that the calculated isotherms, isentropes, and isobars in Figure 3 and Figure 4 demonstrate the normal behavior (monotone increase) of pressure and specific internal energy with increasing absolute temperature and specific entropy at a constant density (at a constant specific volume).

Moreover, the pressure values increase with increasing density (decreasing specific volume) both at a constant temperature (along the shown sections of the isotherms at T=9, 18, 27, and 36 kK) and at a constant specific entropy (along the shown sections of the isentropes at S=0.616, 0.832, 1.008, and 1.171 J/[g K]). Such behavior indicates the stability of the thermodynamic equilibrium states [32] of matter in this region of densities, pressures, and specific internal energies at high temperatures and high specific entropies.

The results of calculations of isotherms and isentropes at relatively low temperatures and low specific entropies in the region of expansion of condensed matter display a drop in pressure with a decrease in density to a certain value (which depends on temperature or entropy), an increase in pressure with further expansion to the next certain density value, and again a drop in pressure with a further increase in specific volume.

The geometrical locus of the points of minimum and maximum pressure in relation to density (or specific volume) on the isotherms corresponds to the boundary of loss of stability of thermodynamic equilibrium states [32]. For the presented equation of state for europium, this boundary is located in the region of density below 5.5 g/cm^3^, pressure below 1 GPa, and temperature below 2.3 kK. A more detailed analysis of this boundary is beyond the scope of this communication.

The behavior of the thermal parts of pressure and specific internal energy ($P_{t}$ and $E_{t}$) on the considered shock adiabats, isotherms, isentropes, and isobars is illustrated by the diagrams of state for europium in Figure 5 and Figure 6. Here, $P_{t}\left(V,E\right)=P\left(V,E\right)-P_{c}\left(V\right)$ and $E_{t}\left(V,E\right)=E-E_{c}\left(V\right)$. These values remain positive throughout the entire range of positive temperatures.

The range of variation of the dimensionless parameter $\xi_{t}$ (12) in the region of thermodynamic parameters under consideration is demonstrated in the diagram of state for the material in Figure 7. It should be emphasized that this range is limited by positive values of the parameter $\xi_{t}$ significantly less than unity, which may prove to be convenient when using the proposed equation of state in practical calculations.

The calculated shock adiabats and the room-temperature isotherm in the coordinate planes $P_{t}$–ρ, $E_{t}$–ρ, and $\xi_{t}$–ρ shown in Figure 5, Figure 6 and Figure 7 are in good agreement with the points corresponding to the data from high-pressure experiments on the shock-wave loading [22,23,24,25] (above 20 GPa) and isothermal compression [31] (for a monoclinic structure) of europium.

The values of the constants of the equation of state for europium within the framework of the presented model were chosen based on the condition of optimal description of the available experimental data as follows: $V_{0}=0.13089$ cm^3^/g, $V_{0c}=0.126499$ cm^3^/g, $B_{0c}=14.4638$ GPa, m=1.7, n=1.63, $\sigma_{m}=0.8$, $\delta_{n}=1$, $\gamma_{0c}=1.2$, $\gamma_{i}=0.45$, $E_{a}=50$ kJ/g, and $\beta_{a}=48.5$. In the calculations, it was assumed that $A_{u}=151.964$ [50].

## 4. Conclusions

Thus, a new model is proposed to describe the thermodynamics of metallic materials based on the characteristic function of specific entropy upon the variables of specific volume and specific internal energy. The equation of state constructed for europium within the framework of this model is consistent with the available data from experiments at high pressures and specific entropies. This equation of state is expressed in a rather simple form and can be effectively used in modeling and simulations of dynamic processes in materials and structures at high energy densities.

## Data Availability

The original contributions presented in this study are included in this communication. Further inquiries can be directed to the author.

## References

[1] Zel’dovich Y.B., Raizer Y.P. (1967). Physics of Shock Waves and High-Temperature Hydrodynamic Phenomena.

[2] Eliezer S., Ghatak A.K., Hora H. (1986). An Introduction to Equations of State: Theory and Applications.

[3] Bushman A.V., Fortov V.E., Kanel’ G.I., Ni A.L. (1993). Intense Dynamic Loading of Condensed Matter.

[4] Lomonosov I.V., Fortova S.V. (2017). Wide-range semiempirical equations of state of matter for numerical simulation on high-energy processes. High Temp..

[5] Zhukov V.P., Bulgakova N.M. (2024). Volumetric modification of transparent materials with two-color laser irradiation: Insight from numerical modeling. Materials.

[6] Fan R., Lin G., Zhang H., Zhang L., Sun W. (2025). Dynamic behavior of submerged cylindrical shells under combined underwater explosion, bubble pulsation, and hydrostatic pressure. Materials.

[7] Zhu W., Yao W., Liu J., Zheng Y., Li W., Wang X. (2025). Numerical investigation on the performance of compressible fluid systems in mitigating close-field blast effects on a fiber circle. Materials.

[8] Zhu W., Li W., Li W., Wang X., Yao W. (2025). Shock response characteristics and equation of state of high-mass-fraction pressed tungsten powder/polytetrafluoroethylene-based composites. Polymers.

[9] Jin Z., Xu W., Yin C., Lei Z., Kong X. (2025). Correction method for initial conditions of underwater explosion. J. Mar. Sci. Eng..

[10] Alharbi M., Alshammari F. (2025). Numerical evaluation of equation of state sensitivity in energy conversion systems. Mathematics.

[11] Luo H., Xin Q., Yao C., Li C., Yang T., Wu X., Chahine R., Xiao J. (2025). Effect of real gas equations on calculation accuracy of thermodynamic state in hydrogen storage tank. Appl. Sci..

[12] Barengolts S.A., Uimanov I.V., Oreshkin V.I., Khishchenko K.V., Oreshkin E.V. (2026). Formation of the surface microrelief of copper electrodes during radiofrequency vacuum breakdown. Vacuum.

[13] Grikshtas R., Efimov S., Asmedianov N., Krasik Y.E. (2026). Underwater electrical explosions of different metal wires on the microsecond timescale. Plasma.

[14] Leinweber G., Barry D.P., Block R.C., Rapp M.J., Hoole J.G., Danon Y., Bahran R.M., Williams D.G., Geuther J.A., Saglime F.J. (2012). Thermal total cross sections of europium from neutron capture and transmission measurements. Trans. Am. Nucl. Soc..

[15] Leinweber G., Barry D.P., Burke J.A., Rapp M.J., Block R.C., Danon Y., Geuther J.A., Saglime F.J. (2014). Europium resonance parameters from neutron capture and transmission measurements in the energy range 0.01–200 eV. Ann. Nucl. Energy.

[16] Lee J., Hori J., Sano T., Nakajima K. (2018). Resonance analysis of ^151,153^Eu from neutron capture cross section measurements in the energy range from 1 to 20 eV. J. Nucl. Sci. Technol..

[17] Mineev V.N., Ivanov A.G. (1976). Electromotive force produced by shock compression of a substance. Sov. Phys. Usp..

[18] Stankus S.V., Khairulin R.A. (1987). Thermal-expansion of europium in the solid and liquid states. High Temp..

[19] Artun O. (2019). Investigation of production of samarium-151 and europium-152,154,155 via particle accelerator. Mod. Phys. Lett. A.

[20] Zhernokletov M.V., Manachkin S.F., Davydov N.B., Raevskii V.A., Blikov A.O., Panov K.N., Ryzhkov A.V., Arinin V.A., Tkachenko B.I., Logvinov A.I. (2023). Quasi-isentropic compression of gaseous helium and deuterium in spherical structures at terapascal pressures. J. Exp. Theor. Phys..

[21] Mochalov M.A., Il’kaev R.I., Erunov S.V., Blikov A.O., Ogorodnikov V.A., Elfimov S.E., Arinin V.A., Komrakov V.A., Likhutov M.I., Maksimkin I.P. (2023). Experimental study of the compressibility of a helium plasma at a pressure up to 20 TPa. JETP Lett..

[22] Bakanova A.A., Dudoladov I.P., Sutulov Y.N. (1970). Electronic transitions in hafnium, europium, and ytterbium at high pressures. Sov. Phys. Solid State.

[23] Gust W.H., Royce E.B. (1973). New electronic interactions in rare-earth metals at high pressure. Phys. Rev. B.

[24] Carter W.J., Fritz J.N., Marsh S.P., McQueen R.G. (1975). Hugoniot equation of state of the lanthanides. J. Phys. Chem. Solids.

[25] Marsh S.P. (1980). LASL Shock Hugoniot Data.

[26] Monfort C.E., Swenson C.A. (1965). The compressions of terbium, europium, scandium and MnSn_2_ to high pressures below 300 °K. J. Phys. Chem. Solids.

[27] Takemura K., Syassen K. (1985). Pressure–volume relations and polymorphism of europium and ytterbium to 30 GPa. J. Phys. F Met. Phys..

[28] Grosshans W.A., Holzapfel W.B. (1985). X-ray studies on europium and ytterbium up to 40 GPa. J. Magn. Magn. Mater..

[29] Bi W., Meng Y., Kumar R.S., Cornelius A.L., Tipton W.W., Hennig R.G., Zhang Y., Chen C., Schilling J.S. (2011). Pressure-induced structural transitions in europium to 92 GPa. Phys. Rev. B.

[30] Husband R.J., Loa I., Munro K.A., McBride E.E., Evans S.R., Liermann H.P., McMahon M.I. (2014). Incommensurate-to-incommensurate phase transition in Eu metal at high pressures. Phys. Rev. B.

[31] Chen B., Tian M., Zhang J., Li B., Xiao Y., Chow P., Kenney-Benson C., Deng H., Zhang J., Sereika R. (2022). Novel valence transition in elemental metal europium around 80 GPa. Phys. Rev. Lett..

[32] Bazarov I.P. (1964). Thermodynamics.

[33] Dolgoborodov A., Rostilov T., Ananev S., Ziborov V., Grishin L., Kuskov M., Zhigach A. (2022). Structure of shock wave in nanoscale porous nickel at pressures up to 7 GPa. Materials.

[34] Rostilov T., Ananev S., Ziborov V., Dolgoborodov A., Vakorina G., Grishin L. (2025). Shock waves in pressed aluminum nanopowder. J. Mech. Phys. Solids.

[35] Kim V.V., Martynenko S.I., Ostrik A.V., Khishchenko K.V., Lomonosov I.V. (2025). Numerical simulation of the initial stage of the formation of the Popigai crater. Dokl. Phys..

[36] He Q.G., Wu D., Yu Y., Zhang H., Wu Q., Hu J. (2025). Generation of spherically converging shock wave based on shock wave lens. Matter Radiat. Extrem..

[37] Semenov A.Y., Abrosimov S.A., Stuchebryukhov I.A., Khishchenko K.V. (2023). Studying the dynamics of wave processes of compression and expansion in palladium under picosecond laser action. High Temp..

[38] Khishchenko K.V., Mayer A.E. (2021). High- and low-entropy layers in solids behind shock and ramp compression waves. Int. J. Mech. Sci..

[39] Yang L., Herbert M.L., Baehtz C., Bouffetier V., Brambrink E., Dornheim T., Fefeu N., Gawne T., Goede S., Hagemann J. (2025). Scaling of thin wire cylindrical compression with material, diameter, and laser energy after 100 fs Joule surface heating. Matter Radiat. Extrem..

[40] Yuan Y., Fan Z., Tu S., Yu C., Miao W., Yang Z., Yin C., Sun L., Du H., Wei M. (2025). Characterizing the evolution of mixing induced by Richtmyer–Meshkov instability in laser-driven reshock experiments. Matter Radiat. Extrem..

[41] Cipriani M., Consoli F., Scisció M., Solovjovas A., Petsi I.A., Malinauskas M., Andreoli P., Cristofari G., Di Ferdinando E., Di Giorgio G. (2026). Experimental and simulation study on high-power laser irradiation of 3D-printed microstructures. Matter Radiat. Extrem..

[42] Khishchenko K.V., Boyarskikh K.A., Obruchkova L.R., Seredkin N.N. (2025). Equation of state for aluminum at high entropies and internal energies in shock waves. Metals.

[43] Bushman A.V., Fortov V.E., Sharipdzhanov I.I. (1977). Equation of state for metals in a wide range of parameters. Teplofiz. Vys. Temp..

[44] Khishchenko K.V. (2023). Equation of state of hafnium at high pressures in shock waves. Phys. Wave Phenom..

[45] Khishchenko K.V. (2023). Equation of state of zirconium at high pressures. High Temp..

[46] Khishchenko K.V. (2024). Equation of state for titanium at high pressures. High Temp..

[47] Landau L.D., Lifshitz E.M. (1980). Statistical Physics.

[48] Tonkov E.Y. (1979). Phase Diagrams of Elements at High Pressures.

[49] Beaudry B.J., Gschneidner K.A., Gschneidner K.A., Eyring L. (1978). Preparation and basic properties of the rare earth metals. Handbook on the Physics and Chemistry of Rare Earths.

[50] Meija J., Coplen T.B., Berglund M., Brand W.A., De Bievre P., Groning M., Holden N.E., Irrgeher J., Loss R.D., Walczyk T. (2016). Atomic weights of the elements 2013 (IUPAC Technical Report). Pure Appl. Chem..

